# Supplementary material for: A microfluidic platform for whole-membrane integrity profiling in live neuronal cells
Source: Microsyst Nanoeng. 2026 Jun 10;12:225. doi: 10.1038/s41378-026-01209-0 (PMC13249814; doi:10.1038/s41378-026-01209-0)
Supplement: Supplementary file 1 — Supplementary Files [file 41378_2026_1209_MOESM1_ESM.docx]

**Table 1:Overview of device performance parameters.** Throughput measurements was determined for a time interval of 30 minutes including measurement of cell rotation.

| Average throughput of cells measured per hour | Maximal throughput per hour | Rate of single cell arrival at cages | Number of sessions per condition | Total experimental  sessions | Average number of experimental sessions per chip |
| --- | --- | --- | --- | --- | --- |
| 20 (With Galton array)  17 (without Galton array) | 30 (With Galton array)  20 (without Galton array) | 0.5-1 cells per min (With Galton array)  0.25-0.66 cells per min (without Galton array) | 3 | 45 | 2.1 |


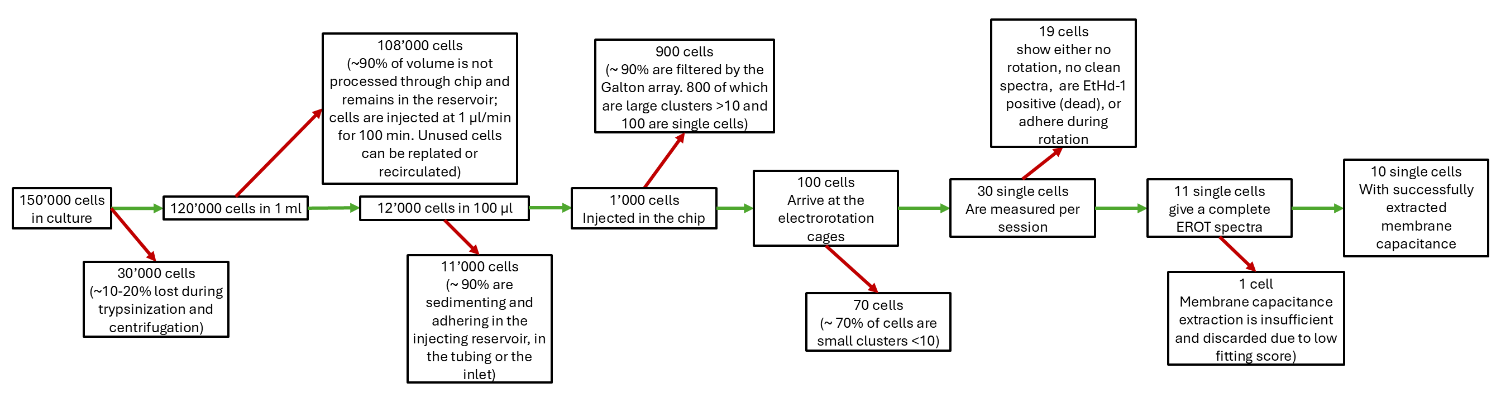
**Fig. S1 Overview of cell yield throughout the measurement workflow.** Reported values represent estimates of the actual device performance obtained across multiple experimental runs. Green arrows indicate cells successfully passing to the subsequent step toward single cell electrorotation measurement, whereas red arrows denote cells lost or deemed unsuitable for experimentation.


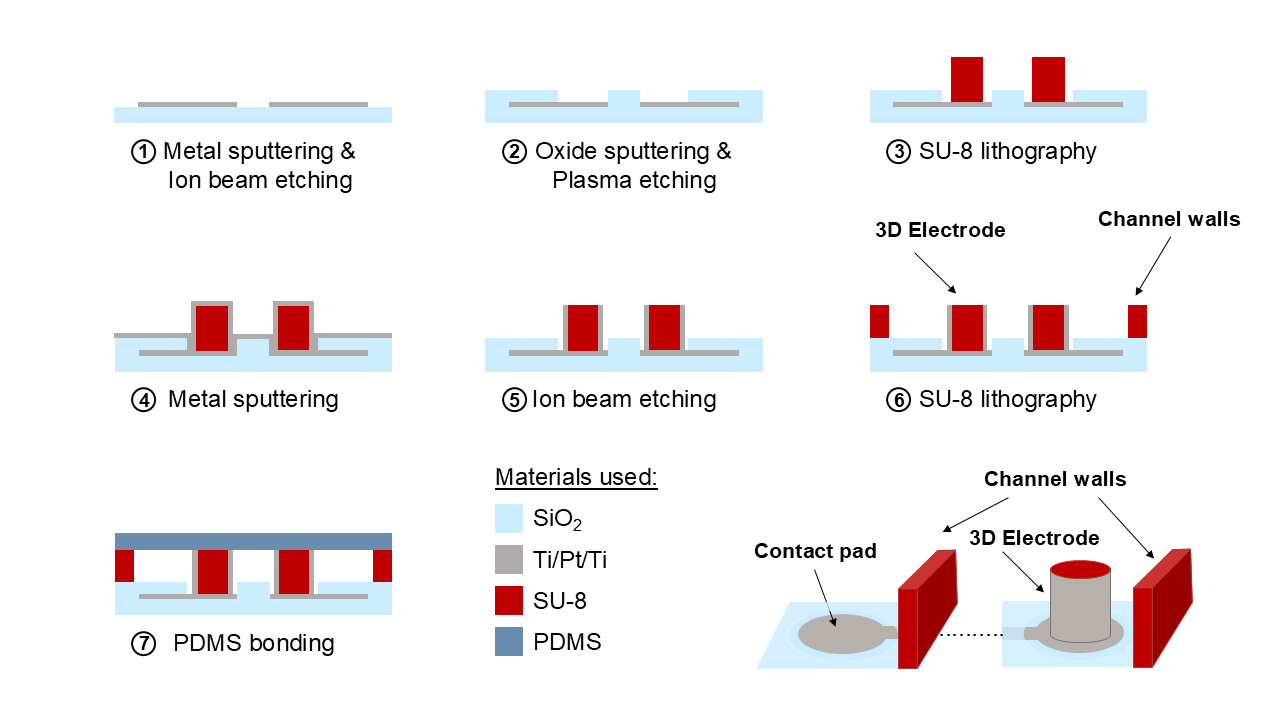
**Fig. S2 Overview of the microfabrication process flow.** Simplified schematic illustration of the device fabrication shown as a 2D cross-section and a three-dimensional rendering. The figure summarizes the key fabrication steps for the formation of the 3D electrodes and the microfluidic channel. Detailed fabrication procedures are described in the Methods.



**Fig. S3 Electrical characterization of on-chip microelectrodes in low-conductivity medium (200 mS/m).** **a** Impedance magnitude and phase for new and reused chips, confirming reusability. **b** −Im(Z) of empty and fluid-filled chips to estimate CDL and CP. **c** Contact pad–to–electrode impedance to determine interconnect resistance.


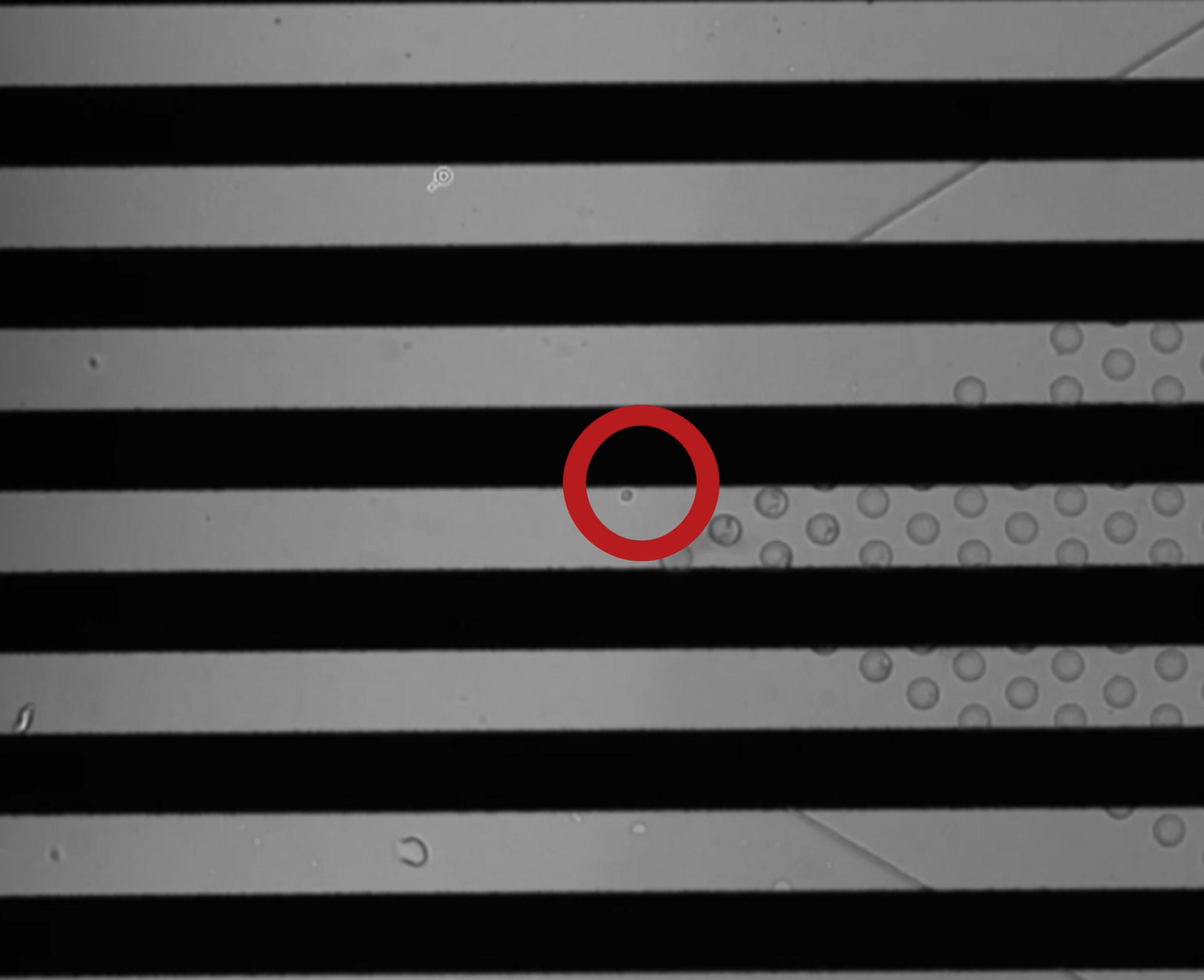


**Supplementary Video 1: Cell passing through the Galton array and finishing in an electrorotation microcage.** Representative video showing a single cell (circled in red) as it passes through the Galton array and is delivered to an electrorotation microcage. Upon arrival at the microcage, the cell is stably trapped and rotated.
